# Rule-Based Pruning and In Silico Identification of Essential Proteins in Yeast PPIN

**DOI:** 10.3390/cells11172648

**Published:** 2022-08-25

**Authors:** Anik Banik, Souvik Podder, Sovan Saha, Piyali Chatterjee, Anup Kumar Halder, Mita Nasipuri, Subhadip Basu, Dariusz Plewczynski

**Affiliations:** 1 Department of Computer Science & Engineering, Dr. Sudhir Chandra Sur Degree Engineering College, 540, Dum Dum Road, Near Dum Dum Jn. Station, Surermath, Kolkata 700074, India; 2 Department of Computer Science & Engineering, Institute of Engineering & Management, Salt Lake Electronics Complex, Kolkata 700091, India; 3 Department of Computer Science & Engineering, Netaji Subhash Engineering College, Techno City, Panchpota, Garia, Kolkata 700152, India; 4 Faculty of Mathematics and Information Sciences, Warsaw University of Technology, Koszykowa 75, 00-662 Warsaw, Poland; 5 Laboratory of Functional and Structural Genomics, Centre of New Technologies, University of Warsaw, Banacha 2c Street, 02-097 Warsaw, Poland; 6 Department of Computer Science & Engineering, Jadavpur University, 188, Raja S.C. Mallick Road, Kolkata 700032, India

**Keywords:** essential protein, edge weight, node weight, yeast PPIN, local interaction density

## Abstract

Proteins are vital for the significant cellular activities of living organisms. However, not all of them are essential. Identifying essential proteins through different biological experiments is relatively more laborious and time-consuming than the computational approaches used in recent times. However, practical implementation of conventional scientific methods sometimes becomes challenging due to poor performance impact in specific scenarios. Thus, more developed and efficient computational prediction models are required for essential protein identification. An effective methodology is proposed in this research, capable of predicting essential proteins in a refined yeast protein–protein interaction network (PPIN). The rule-based refinement is done using protein complex and local interaction density information derived from the neighborhood properties of proteins in the network. Identification and pruning of non-essential proteins are equally crucial here. In the initial phase, careful assessment is performed by applying node and edge weights to identify and discard the non-essential proteins from the interaction network. Three cut-off levels are considered for each node and edge weight for pruning the non-essential proteins. Once the PPIN has been filtered out, the second phase starts with two centralities-based approaches: (1) local interaction density (LID) and (2) local interaction density with protein complex (LIDC), which are successively implemented to identify the essential proteins in the yeast PPIN. Our proposed methodology achieves better performance in comparison to the existing state-of-the-art techniques.

## 1. Introduction

Various research areas like protein structure prediction [1,2]; protein function prediction using protein sequences [3,4], protein domains [5,6], and protein–protein interaction networks (PPIN) [7,8,9,10,11]; protein subcellular localization identification [12,13]; and detection of essential proteins [14,15,16] have significantly been exploited due to the increase in the availability of a large number of proteins/protein sequences in the post-genomic era. In general, essential proteins are the highly connected modules in a PPIN [17]. So, removing any essential protein from the existing network would be fatal, resulting in various functional disorders of living organisms. Most of the research works [18,19,20] note the fact that deeper analyses of essential proteins in a PPIN will lead to better assimilation of ideas about the mutation of genes, which is usually considered as the ultimate cause of disease initiation. Thus, essential protein prediction has a significant role in the medical and biological fields of study. Though computational approaches have become the recent trend for establishing the topological relationship between a PPIN and the essentiality of proteins, the previous biological methodologies [21,22] provided the base for the foundation for this research field. Being directed by the centrality–lethality rule [17], centrality measures based on the topological features of biological PPINs have become the center of attraction for most of the existing methodologies [17,23,24] for the identification of essential proteins.

According to Luo et al. [23], computational approaches to essential protein prediction can be broadly classified into two categories: (1) Topological centrality-based approaches at the PPIN level: Centrality measures derived from the topological properties of a PPIN are considered in the topological centrality-based approach. In the work of Li et al. [15], each protein in a PPIN is represented as a material particle. The author estimated the value of each of these particles’ topology potential, which gave them a unique ranking. Based on these rankings, the essentiality of proteins is derived. Tang et al. [24] developed a Cytoscape [25] plugin named CytoNCA to evaluate biological PPINs through the computation of various centrality scores. Currently, it supports eight centralities for both unweighted and weighted PPINs: betweenness centrality (BC) [26], closeness centrality (CC) [27], degree centrality (DC) [17], eigenvector centrality (EC) [28], local average connectivity-based method (LAC) [29], network centrality (NC) [14], subgraph centrality (SC) [30], and information centrality (IC) [31]. (2) Heterogeneous feature-based approach: The use of topological centrality measures along with protein-specific features is usually considered a heterogeneous feature-based approach. This can be accomplished by incorporating the gene ontology (GO) terms of proteins [32], protein complexes [33,34], orthologous information [35], subcellular protein localization [36], and gene expression data [37,38,39] along with a PPIN. Another recent work by Dong et al. [40] considers five relevant features after reviewing several related features in this field of essential protein prediction: (1) domain information [41,42], (2) evolutionary conservation [43,44], (3) sequence components [45,46], (4) network topology [14,33], and (5) expression level [47,48] for essential protein/gene prediction. They have used a support vector machine (SVM) for the same task after splitting the yeast and human data into train and test sets.

Existing computational approaches reveal a relation between protein degree and essentiality. Nevertheless, some experimental analyses, like yeast two-hybrid (Y2H) analyses, have also created conflict, stating that this association may be too fragile for binary or transient PPINs [49,50]. Modular essentiality is highlighted in the work of Ryan et al. [51], where all the proteins in a protein complex are considered to be essential. In contrast, Wang et al. [52] established a strong foundation indicating that essential proteins do have a more significant number of protein complex interactions. They also stated that larger protein complexes are more likely to become essential than smaller ones. Various researchers [53,54] have also shown that essential proteins are usually present in the denser sub-modules of a PPIN formed by a single protein interacting with its adjacent neighbors to perform a specific biological function. Hence, the relation between protein complexes and essentiality must also be considered. In the work of Hart et al. [55], a scoring method is proposed that can yield a subset of observed matrix-model interactions having high confidence scores. Later, these sets are used to infer a yeast’s most accurate mapping of protein complexes. The results generated from the proposed work of Hart et al. also established that essentiality depends on a protein complex rather than an individual protein. Ren et al. [33] introduced a centrality-based approach, ECC, which is based on SC [30] and protein complexes. Li et al. [34] also proposed a similar approach to Ren et al., known as united complex centrality (UC). An integrated system of gene expression information and some centralities such as BC [26], PeC [37], DC [17], etc. is used in the work of Zhong et al. [56] for the identification of essential proteins. Other related conventional methodologies in this field of study are range-limited centrality [57], L-index [58], coexpression weighted by clustering coefficient (CoEWC) [59], LeaderRank [60], weighted degree centrality (WDC) [61], an iteration method for predicting essential proteins by integrating orthology with a PPI network (ION) [35], and normalized *α*-centrality [62]. Among the previously discussed methodologies of essential protein function prediction, a few important ones are highlighted in Table 1.

Though the existing computational approaches can identify essential proteins efficiently, these methods produce more false positives. To overcome this, a new methodology for essential protein identification is proposed in this work. This method works in two phases: (1) the first phase deals with the non-essential proteins present in the PPIN using two topological features, node and edge weight [64], which ensure the presence of only the reliable nodes and edges in the PPIN—in other words, they focus only on the densely connected modules in the PPIN [7]. (2) In the next phase, local interaction density (LID) [23] and local interaction density with protein complex (LIDC) [23] are used for the identification of essential proteins in the PPIN. All the required data supporting the proposed methodology, including basic terminologies like node weight, edge weight, LID, and LIDC centralities, are given in the Supplementary Materials, available online: https://drive.google.com/drive/folders/1nH3bjxTscorRunDOEAnZT2BXzHXWmRKd?usp=sharing, accessed on 18 August 2022.

In the upcoming section, the dataset of *Yeast* PPIN used for the proposed methodology will be discussed. Following that, the detailed implementation of our rule-based pruning research and the application of LID and LIDC will be highlighted, along with the pictorial representation of PPIN-related terminologies. Finally, the paper will be ended with a results and discussion section, followed by the conclusion.

## 2. Dataset

For the proposed work, the PPIN database of yeast, i.e., *Saccharomyces cerevisiae*, is used. It was downloaded from the DIP database [65,66] (named YDIP_5093 in the work of Luo et al. [23]), which includes 5093 proteins and 24,743 interactions. The PPIN of yeast is highlighted in Figure S1 in Supplementary Materials. Moreover, a protein complex, marked as Complex_745 [23], is also used along with LIDC [23] in the second phase of our proposed methodology. It contains about 745 protein complexes involving 2167 proteins. This protein complex is a combination of four natural protein complex datasets: (1) CM270 is obtained from the MIPS database [67]; (2) CM425 [68] is obtained from MIPS (Mewes 2005), Aloy et al. [69], and the SGD database [70]; (3) the last two, CYC408 and CYC428, are obtained from CYC2008 of the Wodak Laboratory [71,72].

## 3. Methodology

This section proposes a methodology that identifies proteins as topologically more connected by applying a network-based scoring technique to the processed and rule-based pruned network. The network is pruned by removing some nodes and edges having less node weight and edge weight than the specified cut-off value. Thus, less interconnected proteins are identified based on their degree and other parameters and removed, as they are not very topologically significant. The entire working mechanism of the proposed methodology in this research work is highlighted in Algorithm 1.

The PPIN of yeast contains some topologically less important proteins, i.e., proteins having degree 0 or 1 or fewer interconnections between their neighbors than the rest of the proteins, representing their non-essentiality. Edge reliability is another factor that must be considered for identifying essential proteins. Thus, the reliability of every node and edge is investigated by calculating node and edge weights [64] in the first phase of the proposed methodology. The node weight $W_{v}$ of a node v ∈ V in PPI networks [64] is the average degree of all nodes in $G_{v}^{\prime }$, a sub-graph of the network $G_{v}$. It is represented by

$$W_{v}=\sum_{u\in V^{\prime \prime }\;}deg\left(u\right)/\left|V^{\prime \prime }\right|\;$$

where $V^{\prime \prime }\;$ is the set of nodes in $G_{v}^{\prime }$. |$\;V^{\prime \prime }$| is the number of nodes in $\;G_{v}^{\prime }$, and deg(u) is the degree of a node u ϵ $V^{\prime \prime \;}$ in $W_{v}$. The edge weight $W_{uv}\;$ [64] of nodes u and v is represented by

$$W_{uv}=\left(\Gamma \left(u\right)\;\cap \;\Gamma \left(v\right)\right)/\left(\Gamma \left(u\right)\;\cup \;\Gamma \left(v\right)\right)$$

where Γ (u) and Γ (v) are neighbors of u and v, respectively. Γ (u) ∩ Γ (v) represents all common neighbors of u and v, and Γ (u) ∪ Γ (v) means all distinct neighbors of u and v.

Less reliable nodes and interconnections are pruned. Thus, in an interaction network, a protein’s interconnectivity with other proteins and the reliability of those interactions make the pruning strategy stronger. Moreover, setting various cut-off levels for node and edge weights is integral to this phase. So, three cut-off levels, i.e., high, medium, and low [73] (see Algorithm 1), are evaluated to see the changes in the prediction accuracy level in the second phase of essential protein identification. The cut-off ($\theta_{k}$) is calculated by the following mathematical equation:

$$\theta_{k}=\alpha +k\times \sigma \times \left(1-\frac{1}{1+\sigma^{2}}\;\right)$$

where k ∈ {1, 2, 3} defines low, medium, and high cut-offs, respectively. α is determined to be the mean of the node weight/edge weight values, while σ is considered to be the standard deviation of the node weight/edge weight values.

**Figure 1:**
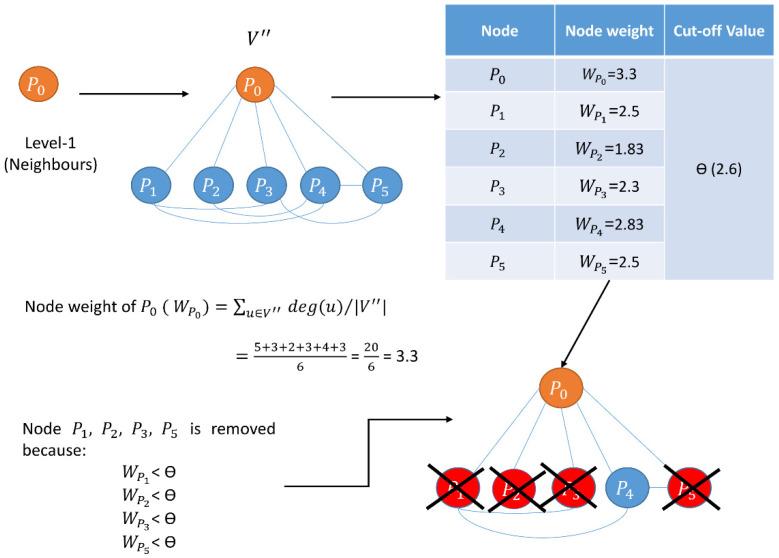
Schematic diagram of computation of node weight. It retains proteins having maximum connectivity. Root node (protein) is denoted by orange while its corresponding neighbors (proteins) are highlighted in blue. The filtered-out nodes (proteins) are represented in red.

**Figure 2:**
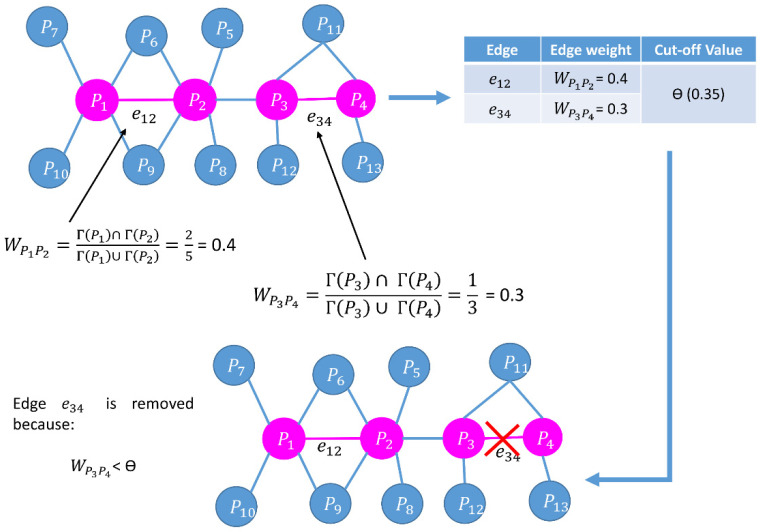
Schematic diagram of computation of edge weight. Edge weight retains only the reliable edges in a PPIN. Edge weight has been calculated for the edges connected with the nodes (proteins) marked with pink color whereas the neighbors (proteins) and their connected edges are highlighted in blue color.

This approach filters out a refined PPIN of yeast containing denser sub-modules [7]. Moreover, as discussed in the introduction, essential proteins tend to lie in the denser sub-modules or protein complexes of a PPIN. Thus, the first phase plays a significant role in this research. The computation of the node and edge weights of two different synthetic networks are highlighted in Figure 1 and Figure 2, respectively.

**Figure 3:**
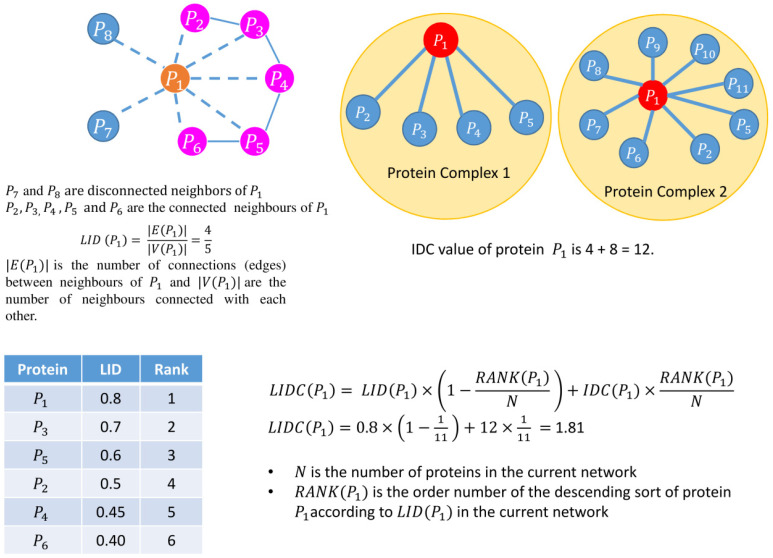
Schematic diagram of computation of LIDC. It is a combination of 3 scores: (1) LID, (2) IDC, and (3) ranking score. Disconnected neighbors (proteins) are highlighted in blue color whereas inter-connected neighbors (proteins) are represented in pink color. Protein complex is represented in yellow.

As discussed in the introduction, computational approaches to essential protein prediction can be of two types: (1) topological centrality-based approaches and (2) heterogeneous feature-based approaches. Experimental data [23] show the topology network centrality-based scoring technique, LID [23], and the heterogeneous feature-based approach, LIDC [23], perform better than the other existing approaches to essential protein identification. So, for each node and edge weight cut-off level in the second phase, LID (Luo and Qi 2015) and LIDC [23] are computed for each protein. LIDC combines heterogeneous values obtained from LID, in-degree centrality of complex (IDC) derived from protein complex Complex_745 [23], and ranking of an individual protein. The procedure for computing LIDC is shown in Figure 3. Finally, the proteins are sorted in descending order according to their computed LIDC values. Protein sets are selected as essential in two different ranking ranges (top 100–200 proteins). This selection strategy is the same as in Luo et al.’s work [23].
**Algorithm1 (Essential Protein Prediction)**Input:    PPIN of yeast Output: List of Essential and Non-essential ProteinBegin //**calculating node weight**
for every node P in the network  Calculate the node weight, $W_{p}=\frac{\sum_{u\in V^{\prime }}\left(\deg\left(u\right)\right)}{\left|V^{\prime }\right|}$
 //$V^{\prime }$ is the set of neighbors of node P, and $\left|V^{\prime }\right|$ is the number of proteins in $V^{\prime }$
 //deg(u) is the degree of a node $u\in V^{\prime }$ //**end of calculating node weight** Compute $\theta_{k}=\alpha +k\times \sigma \times \left(1-\frac{1}{1+\sigma^{2}}\;\right)$// **Cut-off calculation of node weight** //α is the mean of node weight, σ is the standard deviation of node weight, k ∈{1,2,3} denotes three different //cut-offs, i.e., low, medium, and high, respectively. //**reduction of network based on**
$Th_{k}$
**of node weights** for every node P in the network  if node weight of $P<\theta_{k}$
  remove P from the network //**end of reduction of network based on**
$Th_{k}$
**of node weights** //**edge weight calculation** for every edge E in the network  Calculate edge weight, $W_{uv}=\frac{\left|\Gamma \left(u\right)\;\cap \;\Gamma \left(v\right)\right|}{\left|\Gamma \left(u\right)\;\cup \;\Gamma \left(v\right)\right|}$
 //Γ(u) and Γ(v) are the neighbors of u and v, respectively  //*Γ*(*u*) ∩ *Γ*(*v*) represents all common neighbors of *u* and *v*
 //*Γ*(*u*) ∪ *Γ*(*v*) represents all distinct neighbors of *u* and *v* //**end of edge weight calculation** Compute $\theta_{k}=\alpha +k\times \sigma \times \left(1-\frac{1}{1+\sigma^{2}}\right)$//**Cut-off calculation of edge weight** //α is the mean of edge weight, σ is the standard deviation of edge weight, k ∈{1,2,3} denotes three different //cut-offs, i.e., low, medium, and high, respectively. //**reduction of network based on**
$Th_{k}$
**of edge weights** for every edge E in the network  if edge weight of $E<\theta_{k}$
  remove E from the network //**end of reduction of network based on**
$Th_{k}$
**of edge weights** //**calculate LIDC for low, medium, and high node edge weight** //**calculation of LIDC** for every node u in the pruned network, compute  $LID\left(u\right)=\frac{\left|E\left(u\right)\right|}{\left|V\left(u\right)\right|}$
 //|E(u)| is the number of connections (edges) between neighbors of u, and |V(u)| are the number of neighbors  //connected with each other  //**end of calculation of LID** $IDC\left(u\right)=\underset{i\in ComplexSet\left(u\right)}{\sum }IN-Degree{\left(u\right)}_{i}\;$
 //ComplexSet(u) denotes a set of protein complexes that include protein u
 //$IN-Degree{\left(u\right)}_{i}$ is the degree of protein u in ith protein complex that belongs to ComplexSet(u)
 //**end of calculation of IDC**
 $LIDC\left(u\right)=LID\left(u\right)\times \left(1-\frac{RANK\left(u\right)}{N}\;\right)+IDC\left(u\right)\times \frac{RANK\left(u\right)}{N}$
 //LID(u) is the value of the LID, IDC(u) is the value of IDC of the protein complex of protein u,  //N is the number of proteins in the current network,   //RANK(u) is the order number of the descending sort of protein u according to LID(u) in the current network  //**end of calculation of LIDC** Choose proteins in six ranking ranges (top 100–600) as essential protein sets. End

## 4. Result and Discussion

**Figure 4:**
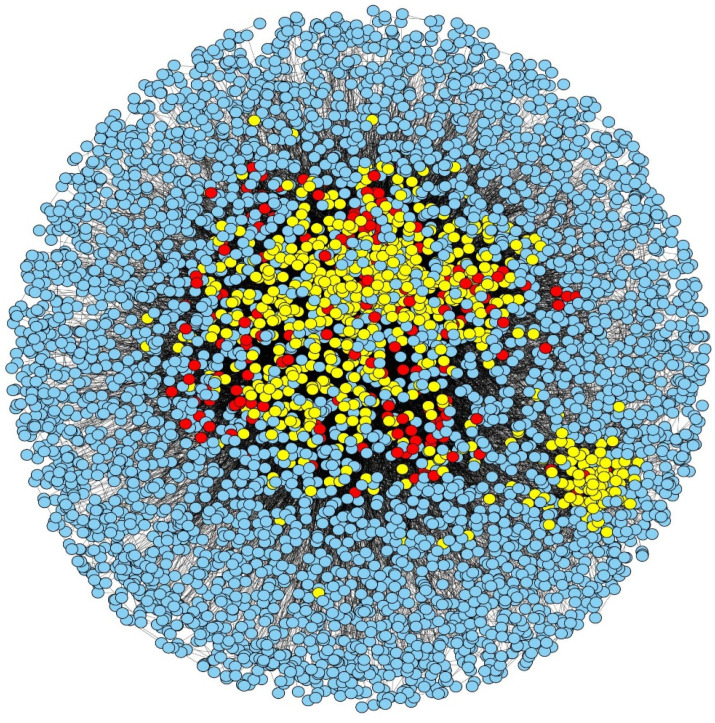
Essential and non-essential proteins in PPIN of yeast at a low cut-off. The yellow-colored proteins are the predicted non-essential ones, while the red ones are the predicted essential proteins. The blue-colored nodes represent proteins that are filtered out in the pre-filtering stage.

**Figure 5:**
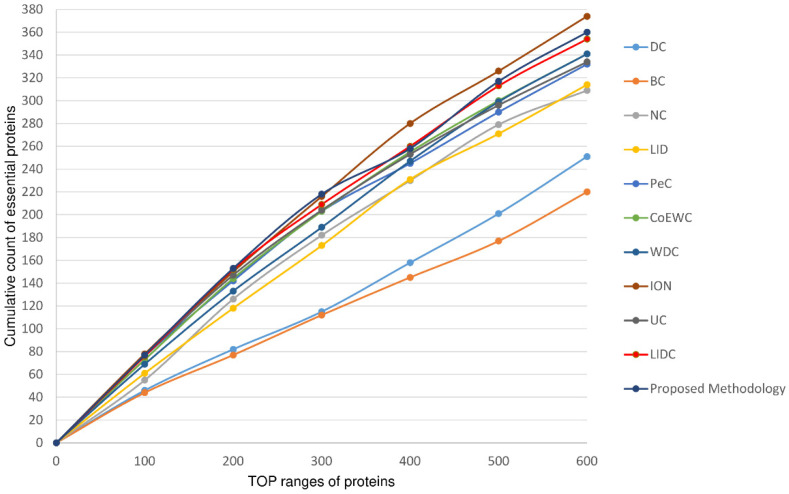
Validation of proposed methodology. All the methods are compared using the jackknife methodology for six different ranking ranges (top 100–600 proteins).

As mentioned earlier, in this proposed work, an LIDC-based scoring technique [23] is used to mark proteins as essential in the topologically processed PPIN, and six different ranking ranges (top 100–600 proteins) are considered. The PPIN of yeast after predicting essential and non-essential proteins at ranking 100 is highlighted in Figure 4. The essentialness of protein sets in the different ranking ranges (top 100–600) at three different cut-offs, i.e., low node and edge weight, medium node and edge weight, and high node and edge weight, are validated against the essential protein set [23] (containing 1285 essential and 4394 non-essential proteins) formed from different databases like MIPS [67], SGD [70], DEG [74], and SGDP [75]. The comparison of the number of predicted essential proteins by our proposed method and several other existing methods like DC [17], BC [26], NC [14], LID [23], PeC [37], CoEWC [59], WDC [61], ION [35], LIDC [23], UC [34], etc. at the three cut-off levels are highlighted in the Supplementary Figures, i.e., Figures S2, S3, and S5–S8. From these figures, it is clear that our method generates an almost equal or greater number of essential proteins compared to LIDC [23] in most cases of the cut-off. This number is comparatively higher when compared to the other methods except for ION. The same observation has also been noted when the jackknife methodology is used to evaluate the proposed method against the others (see Figure 5). Though 20 percent of proteins are considered for evaluating precision, recall, and F-Score, our proposed methodology surpasses the others (see Table 2).

To compare and validate the performance of the proposed method, the top 20 percent of proteins [23] from the ranking result are selected as essential, while the remaining proteins are designated as non-essential. This selection strategy is the same as in Luo et al.’s work [23]. Precision, recall, and F-score are considered performance evaluation metrics. The performance analysis is highlighted in Table 2. It can be derived from Table 2 that our proposed method performs better than the others in terms of precision, recall, and F-score. This signifies that it succeeds in returning most of the relevant proteins compared to the training set of essential proteins. High precision also indicates a low false positive rate. Removing less important nodes and edges and working on the pruned network makes our proposed method worthy and superior to the methods listed in Table 2 and enables us to get high precision, recall, and F-score values.

Our proposed method’s satisfactory performance is achieved using node and edge weights with three proper levels of cut-offs. The pruned PPIN network of yeast at ranking 100 is shown in Figure S4 in the Supplementary Materials. It should also be noted here that though the working mechanisms of LIDC [23] and our proposed method are almost the same, LIDC [23] is applied to the entire PPIN database of yeast, while our proposed method works on a filtered PPIN generated by using three levels of cut-offs on both node and edge weights. The statistics of predicted essential proteins in a filtered PPIN of yeast at three cut-off levels—low node and edge weight, medium node and edge weight, and high node and edge weight—are displayed in Table 3. The overall precision, recall, and F-score at three levels of cut-offs are shown in Table 4.

## 5. Conclusions

Identifying essential proteins is considered one of the most challenging research areas. It helps us identify the significant proteins that are biologically active and play a crucial part in performing vital specific functions of the human body. These proteins might also be essential in transmitting disease or infection when the body is exposed to pathogens. Thus, the computational methods developed for identifying essential proteins should be very effective. PPIN is one of the resources through which this can be done. However, it should be borne in mind that all the network features must be adequately assessed, and the presence of reliable nodes and edges must be ensured. The proposed methodology efficiently identifies essential proteins from a pruned network using local interaction density and local interaction density with a protein complex. The rule-based network pruning is based on specific cut-off edge and node weight values. A detailed comparative study on the performance evaluation of the proposed method and other methods reveals the superiority of this method over others. Because this method solely depends on topological attributes, care should be taken to use a noise-free protein–protein interaction network. This work may be extended to the protein interaction network of any other organism in our future work. However, it should be kept in mind that the essentiality of genes is dynamic. It depends upon the surrounding environment. So, even if several PPIN data of yeast are used for the computational identification of essential proteins/genes, it cannot be assured that the genetic backgrounds set as an experimental environment for all the yeast strains are similar or not [76].

## Figures and Tables

**Table 1:** Computational studies based on essential protein prediction.

Utilized Features	Description	Database	References
Subcellular localization	An efficient method to identify essential proteins for different species by integrating protein subcellular localization information.	PPIN of *Saccharomyces cerevisiae*, *Homo sapiens*, *Mus musculus* and *Drosophila melanogaster*	[36]
Protein complex, degree, subgraph	A new method for predicting essential proteins based on participation degree in protein complex and subgraph Density.	PPIN of *Saccharomyces cerevisiae*	[54]
Orthology, gene expression, PPIN	Predicting essential proteins by integrating orthology, gene expressions, and PPIN.	PPIN of *Saccharomyces cerevisiae*	[39]
CC and orthology	United neighborhood closeness centrality and orthology for predicting essential proteins.	PPIN of *Saccharomyces cerevisiae*	[63]
Node, edge clustering coefficient	Identification of essential proteins using improved node and edge clustering coefficient.	PPIN of *Saccharomyces cerevisiae* and *Drosophila melanogaster*	[22]
Centrality scores	CytoNCA: a cytoscape plugin for centrality analysis and evaluation of protein interaction networks.	_	[24]
Protein complex	Identification of essential proteins based on a new combination of local interaction density and protein complexes.	PPIN of *Saccharomyces cerevisiae*	[23]
PPIN, protein complex	Prediction of essential proteins by integration of PPI network topology and protein complex information.	PPIN of *Saccharomyces cerevisiae*	[33]

**Table 2:** Performance analysis of proposed method with other methodologies.

Methods	Precision	Recall	F-Score
DC (Jeong et al. 2001)	0.41	0.35	0.38
BC (Joy et al. 2005)	0.35	0.31	0.33
NC (Jianxin Wang et al. 2012)	0.46	0.40	0.43
LID (Luo and Qi 2015)	0.45	0.39	0.42
PeC (Li et al. 2012)	0.46	0.40	0.43
CoEWC (Zhang et al. 2013)	0.47	0.41	0.44
WDC (Xiwei et al. 2014)	0.48	0.42	0.45
ION (Peng et al. 2012)	0.53	0.41	0.46
UC (Li et al. 2017)	0.48	0.42	0.45
LIDC (Luo and Qi 2015)	0.50	0.44	0.47
Proposed Methodology	0.77	0.44	0.56

**Table 3:** Network statistics of pruned PPIN of yeast at three levels of cut-offs.

Cut-Off Levels	Proteins after Node Reduction	Interactions after Node Reduction	Proteins after Edge Reduction	Interactions after Node Reduction	Essential Protein	Non-Essential Protein
Low	1393	14,063	985	3907	198	787
Medium	1374	13,924	969	3847	194	775
High	1340	13,714	931	3733	187	744

**Table 4:** Performance analysis of our proposed method at three levels of cut-offs.

Cut-Off Levels	Recall	Precision	F-Score
Low	0.41	0.75	0.53
Medium	0.42	0.76	0.54
High	0.44	0.77	0.56

## Data Availability

The source code of the work is available on GitHub at the following link (https://github.com/SovanSaha/Rule-based-pruning-and-In-Silico-identification-of-essential-proteins-in-Yeast-PPIN.git, accessed on 18 August 2022) for free academic use.

## Supplementary Materials

The following supporting information can be downloaded at: https://www.mdpi.com/article/10.3390/cells11172648/s1. Figure S1: PPIN Network of Yeast of YDIP_5093. It contains 5093 proteins and 24743 interactions; Figure S2: Prediction comparison. Comparison of number of predicted essential proteins for low node and edge weight threshold; Figure S3: Prediction comparison. Comparison of top 100 and top 200 predicted essential proteins for medium node and edge weight threshold; Figure S4: Pruned PPIN of yeast at Low Threshold. Yellow colored nodes are non-essential proteins while the green colored nodes are the essential ones; Figure S5: Prediction comparison. Comparison of top 100 and top 200 predicted essential proteins for high node and edge weight threshold; Figure S6: Prediction comparison. Comparison of number of top 300, top 400, top 500 and top 600 predicted essential proteins for low node and edge weight threshold; Figure S7: Prediction comparison. Comparison of number of top 300, top 400, top 500 and top 600 predicted essential proteins for medium node and edge weight threshold; Figure S8: Prediction comparison. Comparison of number of top 300, top 400, top 500 and top 600 predicted essential proteins for high node and edge weight threshold.
