# Supplementary material for: Rule-Based Pruning and In Silico Identification of Essential Proteins in Yeast PPIN
Source: Cells. 2022 Aug 25;11(17):2648. doi: 10.3390/cells11172648 (PMC9454873; doi:10.3390/cells11172648)
Supplement: Supplementary file 1 [file cells-11-02648-s001.zip › cells-1863857-supplementary.pdf]

# Rule-based pruning and In Silico identification of essential proteins in *Yeast* PPIN : Supplementary Document

Anik Banik[1], Souvik Podder[1], Sovan Saha [2], Piyali Chatterjee[3], Anup Kumar Halder[4], Mita Nasipuri[5] , Subhadip Basu[5], Dariusz Plewczynski[4]

[1]Department of Computer Science & Engineering, Dr. Sudhir Chandra Sur Degree Engineering College, 540, Dum Dum Road, Near Dum Dum Jn. Station, Surermath, Kolkata, 700074, India

[2]Department of Computer Science & Engineering, Institute of Engineering & Management, Salt Lake Electronics Complex, Kolkata – 700091, India.

[3]Department of Computer Science & Engineering, Netaji Subhash Engineering College, Techno City, Panchpota, Garia, Kolkata, 700152, India

[4]Faculty of Mathematics and Information Sciences, Warsaw University of Technology, Koszykowa 75, 00-662 Warsaw, Poland

[4]Laboratory of Functional and Structural Genomics, Centre of New Technologies, University of Warsaw, Banacha 2c Street, 02-097 Warsaw, Poland

[5]Department of Computer Science & Engineering, Jadavpur University, 188, Raja S.C. Mallick Road, Kolkata, 700032, India

**RULE-BASED PRUNING AND IN SILICO IDENTIFICATION OF ESSENTIAL PROTEINS IN *YEAST* PPIN**

**: SUPPLEMENTARY DOCUMENT**

## Basic Terminologies

**Edge weight** ($W_{uv}$)**:** The weight $W_{uv}$ of edge $(u,\ v)$ [1] is defined as the similarity between $u$ and $v$. It is obvious that two nodes with an edge between them belong to the same cluster if they have high similarity. The similarity between $u$ and $v$ is measured by Jaccard's coefficient. Jaccard's coefficient adopts the proportion of common neighbours of two nodes in all distinct neighbours of these nodes to measure node similarity in complex networks. Obviously, the more common neighbours two nodes share, the higher similarity these nodes have. Therefore, the edge weight $W_{uv}$ is represented by

$$W_{uv} = (\Gamma(u)\ \cap\ \Gamma(v)\ )/(\Gamma(u)\ \cup\ \Gamma(v))$$

where $\Gamma(u)$ and $\Gamma(v)$ are neighbours of $u$ and $v$ respectively. $\Gamma(u) \cap \Gamma(v)$ represents all common neighbours of $u$ and $v$, and $\Gamma(u) \cup \Gamma(v)$ represents all distinct neighbours of $u$ and $v$.

**Node weight** ( $W_v$)**:** In $G_v$, there are some nodes with degree 1 that only have connections with $v$ and the connections among these nodes are often false positive according to topological reliability measures [1].So nodes with degree 1 and corresponding edges are removed from $G_v$. The same is also true for nodes having degree 0. The remaining sub graph of $G_v$ is marked as $G'_v$.The node weight $W_v$ of node $v \in V$ in PPI networks[1] is the average degree of all nodes in $G'_v$. It is represented by

$$W_v = \sum_{u \in V''} deg(u)/|V''|$$

where, $V''$ is the set of nodes in $G'_v$.$|\ V''|$ is the number of nodes in $G'_v$. And $deg(u)$ is the degree of a node $u \in V''$ in $W_v$.

**LID centrality:** The LID (Local Interaction Density) of a node $u$ ($LID(u)$)[2] is defined as the density of interactions among its interactive neighbors:

$$LID(u) = |E^{(u)}_{NB\_INT}|/|V^{(u)}_{NB\_INT}|$$

where the operator || is a count of the number of elements in a set, edges in $E^{(u)}_{NB\_INT}$ are called interactive edges while nodes in $V^{(u)}_{NB\_INT}$ are call interactive neighbors.

**LIDC centrality:** The LIDC (Local Interaction Density with Protein Complex)[2] can be computed as:

$$LIDC(u) = LID(u) \times \left(1 - \frac{RANK(u)}{N}\right) + IDC(u) \times \frac{RANK(u)}{N}$$

where $LID(u)$ is the value of the LID, $IDC(u)$ is the value of IDC (Interaction Density of Protein Complex) of the protein complex of protein $u$, $N$ is the number of proteins in the current network, $RANK(u)$ is the order number of the descending sort of protein u according to $LIDC(u)$ in the current network

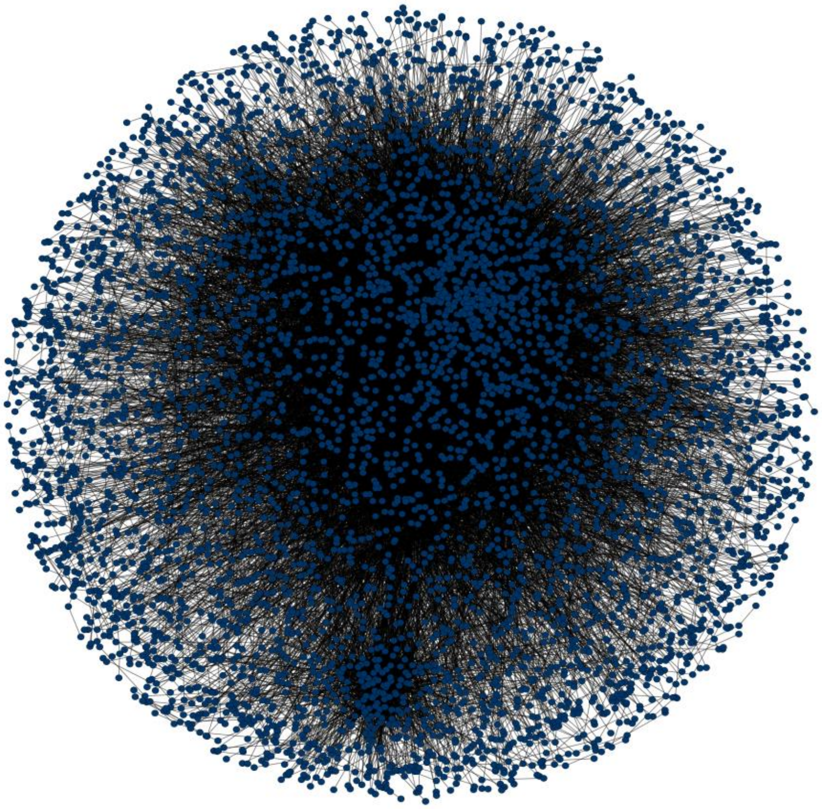

Figure **S1** PPIN Network of Yeast of YDIP_5093. It contains 5093 proteins and 24743 interactions.

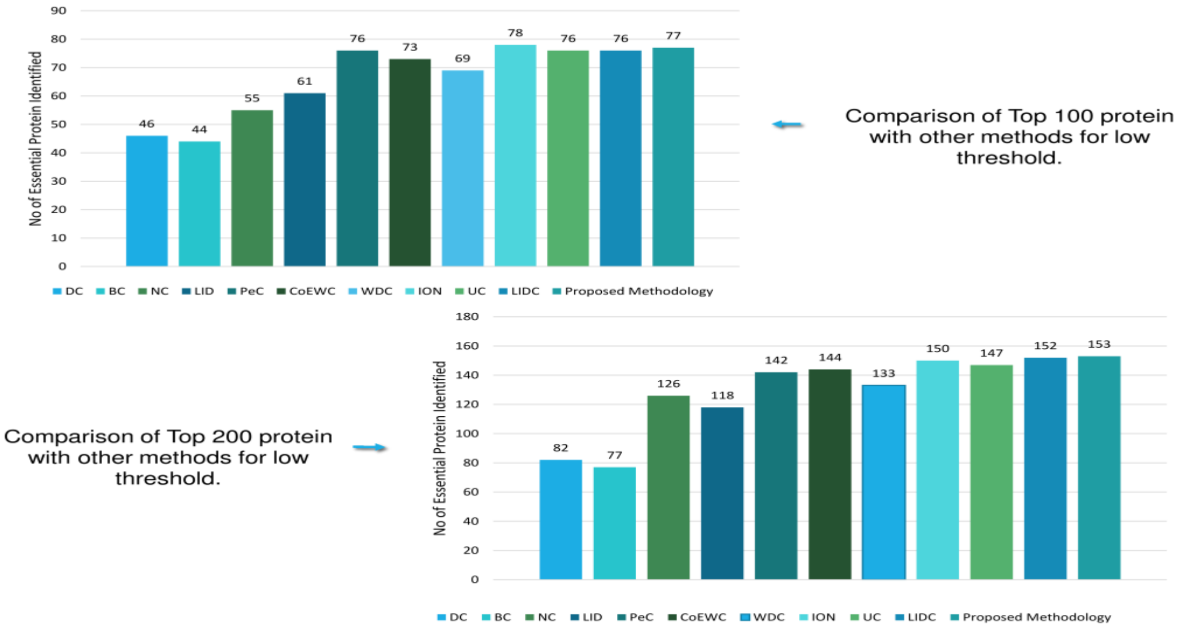


Figure **S2** Prediction comparison. Comparison of number of predicted essential proteins for low node and edge weight threshold.

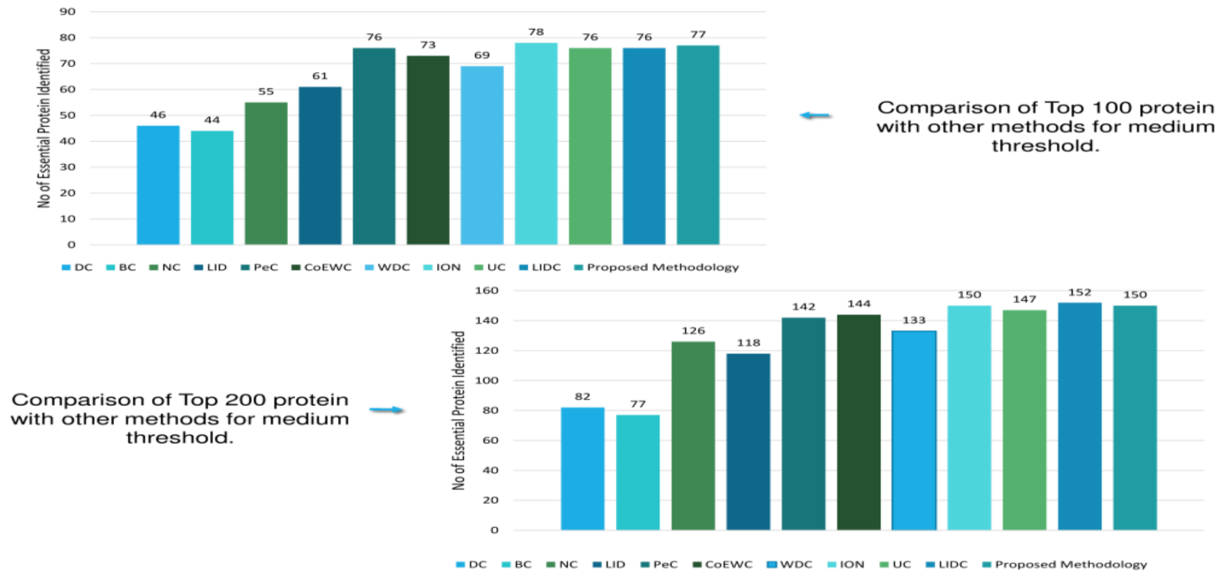


Figure **S3** Prediction comparison. Comparison of top 100 and top 200 predicted essential proteins for medium node and edge weight threshold.

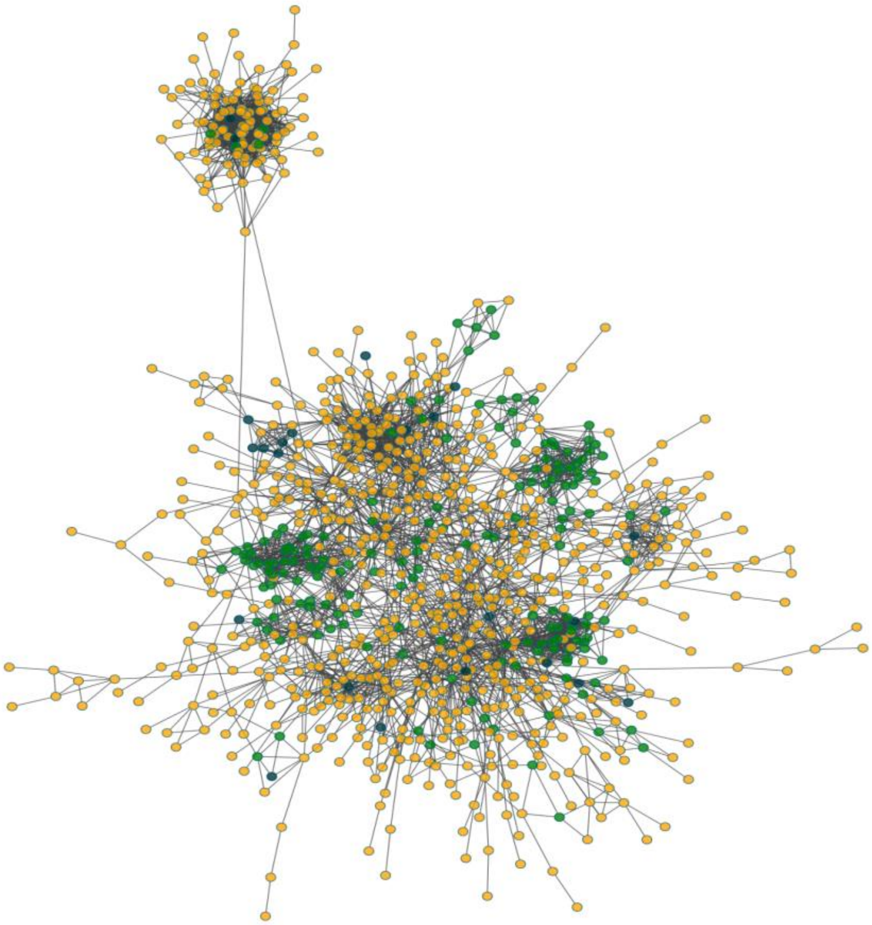

Figure **S4** Pruned PPIN of yeast at Low Threshold. Yellow colored nodes are non-essential proteins while the green colored nodes are the essential ones.

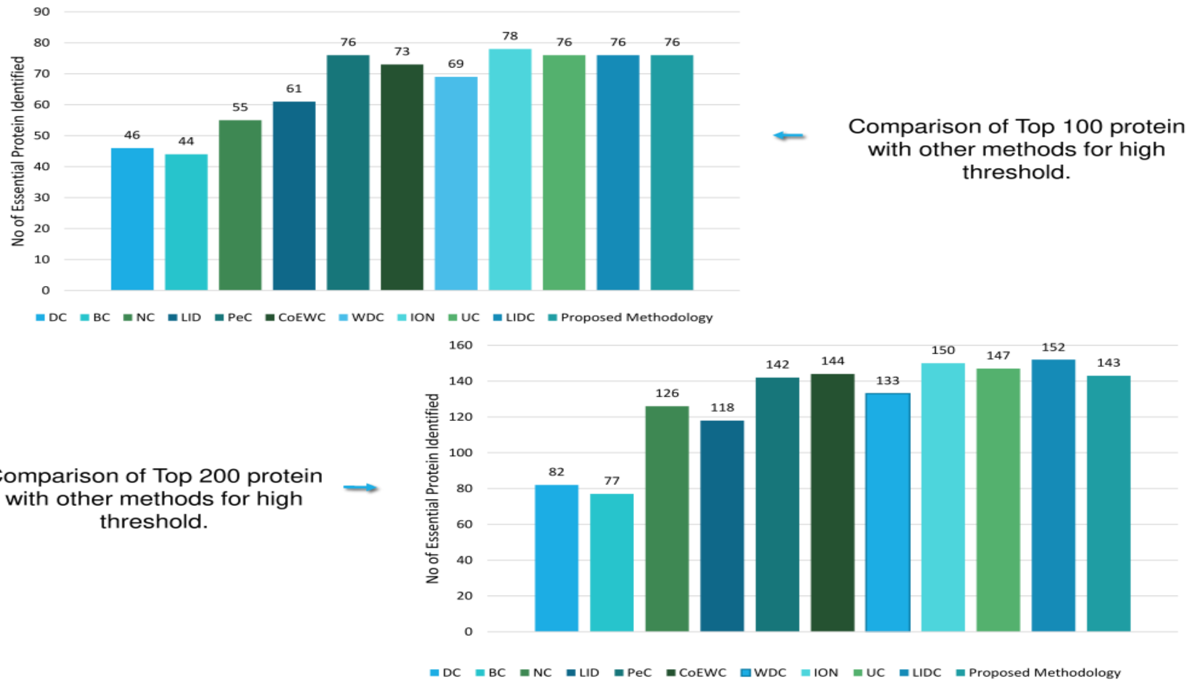


Figure **S5** Prediction comparison. Comparison of top 100 and top 200 predicted essential proteins for high node and edge weight threshold.

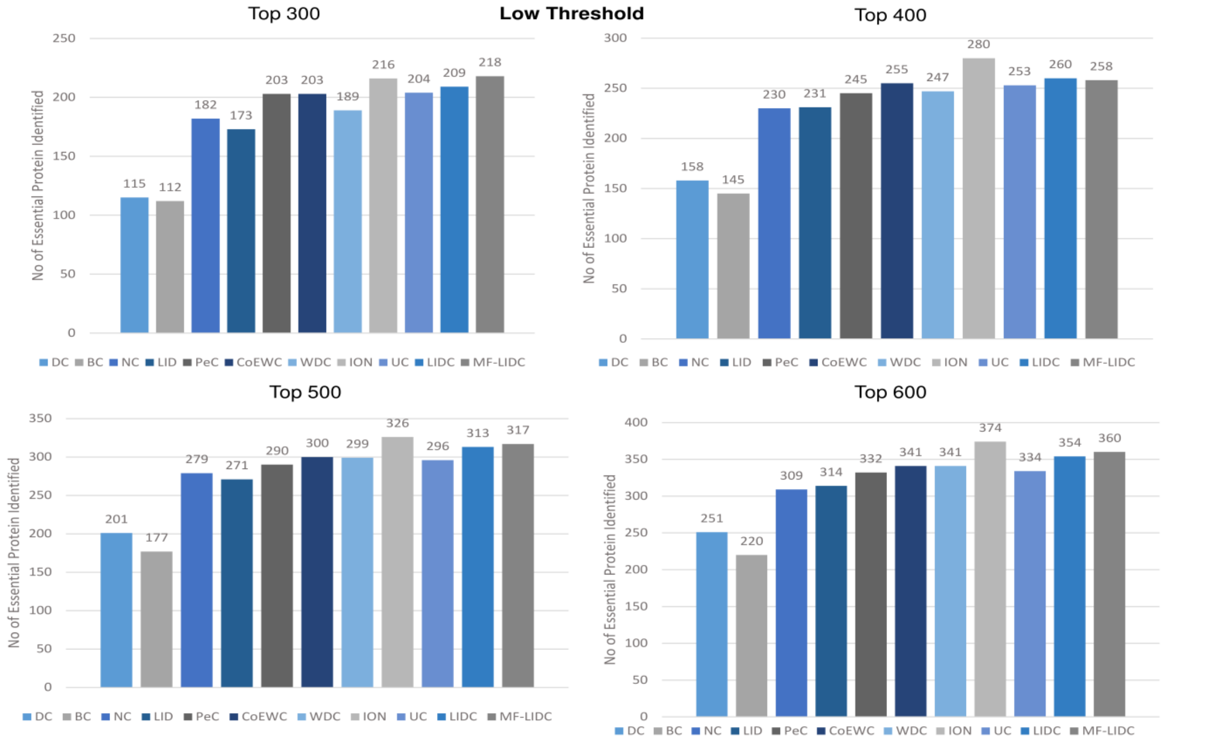


Figure **S6** Prediction comparison. Comparison of number of top 300, top 400, top 500 and top 600 predicted essential proteins for low node and edge weight threshold.

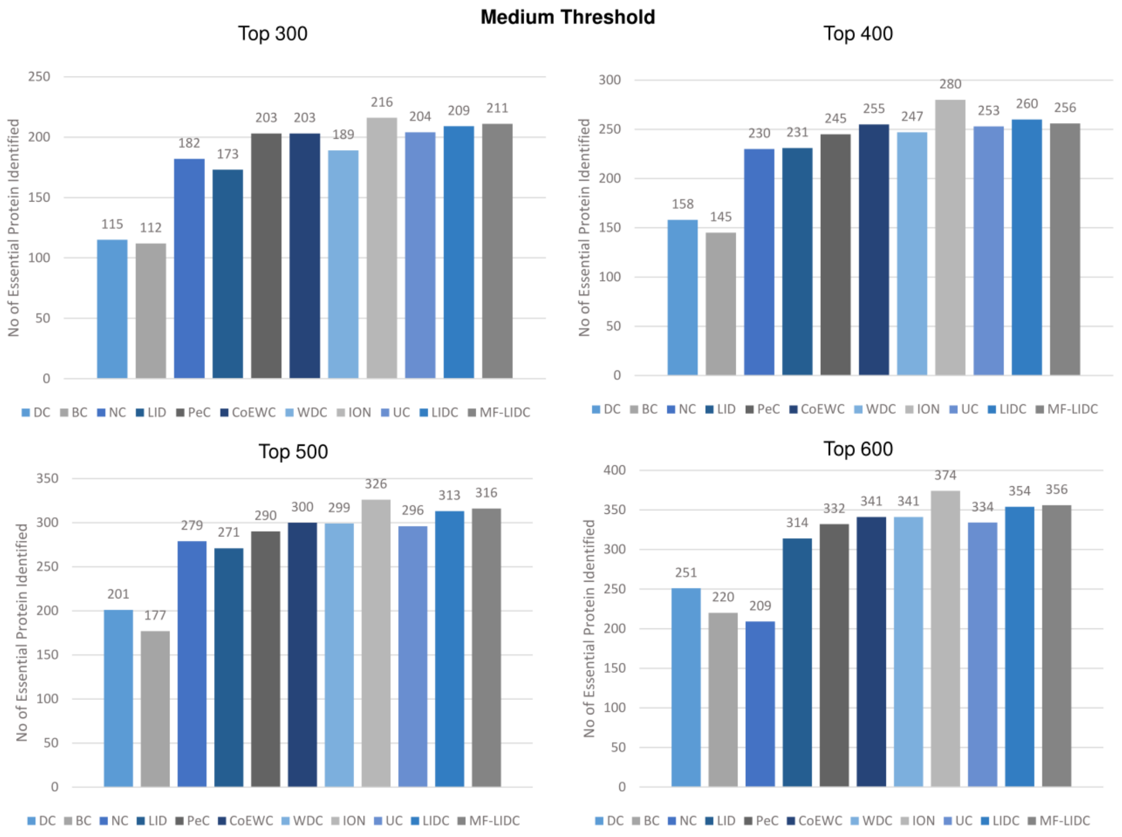


Figure **S7** Prediction comparison. Comparison of number of top 300, top 400, top 500 and top 600 predicted essential proteins for medium node and edge weight threshold.

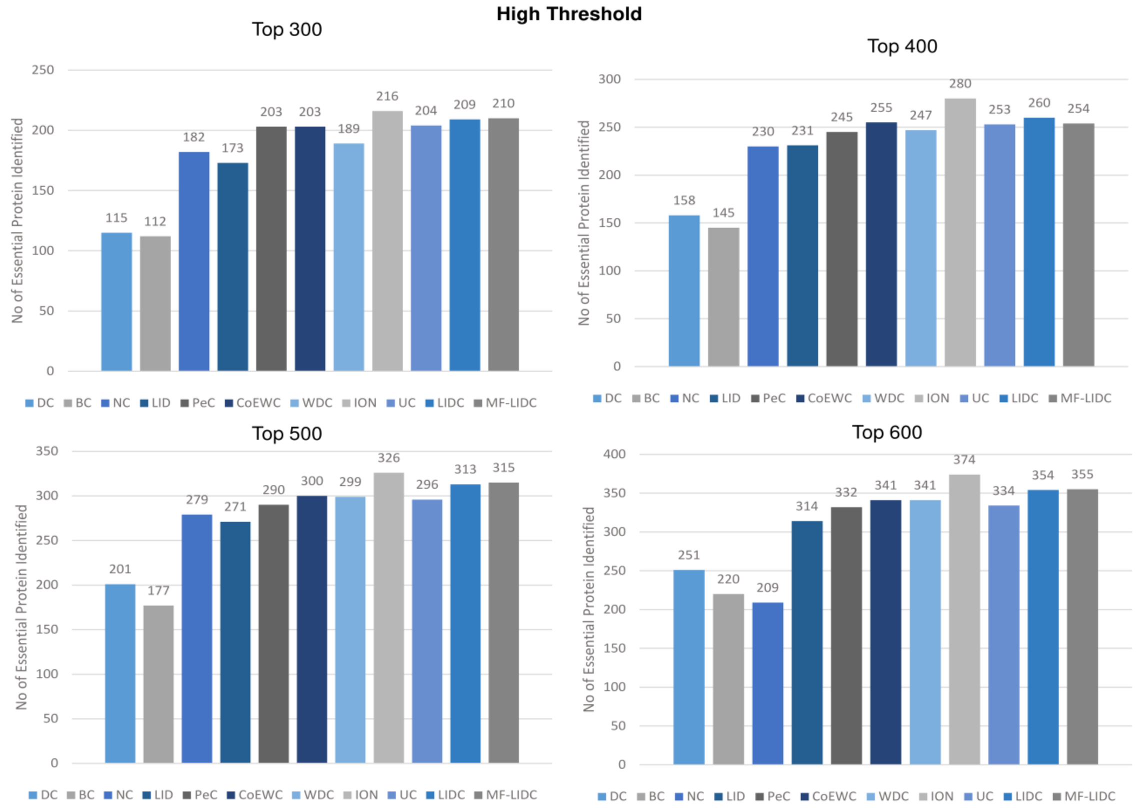


Figure **S8** Prediction comparison. Comparison of number of top 300, top 400, top 500 and top 600 predicted essential proteins for high node and edge weight threshold.

## References

1. Wang, S., and Wu, F. (2013) Detecting overlapping protein complexes in PPI networks based on robustness. Proteome Science **11**, S18-S18.
2. Luo, J., and Qi, Y. (2015) Identification of Essential Proteins Based on a New Combination of Local Interaction Density and Protein Complexes. PLoS ONE **10**, e0131418-e0131418.
